# Potential statin-drug interactions: prevalence and clinical significance

**DOI:** 10.1186/2193-1801-3-168

**Published:** 2014-03-31

**Authors:** Maria Zhelyazkova-Savova, Silvia Gancheva, Vera Sirakova

**Affiliations:** Department of Preclinical and Clinical Pharmacology, Varna Medical University, 55 Marin Drinov Street, Varna, 9002 Bulgaria; University Hospital “St Marina”, First Clinic of Cardiology, 1 Hristo Smirnenski Street, Varna, 9010 Bulgaria

**Keywords:** Statin-drug interactions, Hospital admission, Hospital discharge, CYP inhibitors, Acenocoumarol-statin interactions, INR

## Abstract

**Background:**

Statins are cholesterol-lowering drugs widely used for cardiovascular prevention. Although safe when used alone, in combination with other drugs the likelihood of adverse drug reactions increases significantly. The exposure of the Bulgarian population to coprescriptions leading to potential statin-drug interactions is currently unknown.

**Objective:**

The aim of this study was to investigate the incidence of coprescriptions involving statins and to compare the exposure of outpatients and inpatients to potential statin-drug interactions.

**Setting:**

A cardiology clinic of the teaching University hospital in Varna, Bulgaria.

**Method:**

This observational retrospective study examined the medical records of hospitalized patients prescribed a statin in combination with potentially interacting drugs. Patients who entered the hospital with a statin coprescription (considered outpatients) were compared with those coprescribed a statin at discharge from hospital (considered inpatients). Potentially interacting drugs included inhibitors and inducers of cytochrome P450 (CYP) enzymes and drugs of narrow safety margin (coumarin anticoagulants, digitalis).

**Main outcome measure:**

The proportion of patients exposed to statin coprescriptions with potentially interacting drugs at hospital admission and discharge. Secondary outcome measures: laboratory evidence supporting possible statin-drug interactions.

**Results:**

Out of 1641 hospitalized patients examined, 572 were prescribed a statin, either at hospital admission or discharge. Simvastatin was most commonly prescribed and simvastatin-drug coprescription predominated, especially at discharge. The exposure to all potential statin-drug interactions was similar at hospital admission (26.1%) and discharge (24.4%), as was the exposure to statin combinations with CYP inhibitors, 6.4% and 4%, correspondingly. Overall, more coprescriptions were generated, than were eliminated by hospital physicians. Amiodarone was the CYP inhibitor most frequently coprescribed. Of all interacting drugs acenocoumarol was the most commonly found, the proportions of statin-acenocoumarol coprescriptions being roughly the same at hospital entry (11.5%) and discharge (12.4%). In 7 patients out of 69 exposed to the combination, INR was found to be higher than 3, indicating a risk of over-anticoagulation.

**Conclusions:**

Potential statin-drug interactions are common. Although they do not differ between outpatient and inpatient settings, new hazardous coprescriptions are more frequently generated in hospital. Caution is required when acenocoumarol is coprescribed with statins, especially simvastatin.

## Background

Drug-drug interactions (DDIs) represent an important clinical problem. They substantially increase the rate of adverse drug reactions which may be severe enough to require hospitalization. Up to 2.8% of hospital admissions have been found to result from DDIs (Jankel and Fitterman 1993).

The significance of the problem is often underestimated by physicians and this exposes the patients to the risk of otherwise preventable complications. At the same time, use of more than one drug is often inevitable in the routine clinical practice. Knowledge of the mechanisms and the manifestations of DDIs, as well as their actual incidence and clinical relevance, provides an important tool in avoiding potentially harmful reactions.

Hydroxymethylglutaryl-CoA reductase inhibitors (known as statins) are widely used in the primary and secondary cardiovascular prevention as lipid-lowering drugs. The safety of statins is well recognized; yet possible complications due to their toxicity, albeit rare, should not be overlooked. The risks, especially of myotoxicity, are considerably elevated in combination with other drugs. Since most of the statins are metabolized by cytochrome P450 (CYP) enzyme family, CYP-inhibitors are frequently reported to increase the adverse reactions of statins. Drugs with similar toxicity can also augment the likelihood of muscular and other statin-induced damage. Evidence reveals that over 50% of the statin-associated cases of rhabdomyolysis are secondary to drug interactions (Omar et al. 2001; Bottorff 2006; Bellosta and Corsini 2012).

Potentially hazardous statin-drug interactions (SDIs) can also occur when statins increase the likelihood of other drugs’ toxicity, particularly of drugs with narrow safety margin such as coumarin anticoagulants and digitalis.

The exposure of the Bulgarian population to coprescriptions leading to potential statin-drug interactions is currently unknown.

**The aim** of the present study was to assess the prevalence of hazardous potential SDIs (pSDIs) as a whole and of individual statins. We were also interested to find out whether these coprescriptions were mainly generated in or out of hospital. This is why we checked the statin coprescriptions in hospitalized patients in two separate points in time – at their admission to hospital and at their discharge.

## Methods

This was an observational retrospective study on patients hospitalized from July 2007 to June 2008 in a Cardiology Clinic of the University Hospital of Varna, Bulgaria. The patients’ medical records were retrospectively examined and those receiving a statin at admission or discharge were selected. Patients with a statin prescription at admission were considered outpatients and patients leaving the hospital with a statin prescription were considered inpatients. The medication list, as well as the diagnosis and laboratory results of the patients was carefully registered. Eventually, the patients given (a) potentially interacting drug (s) concomitantly with a statin were selected and further analyzed.

Given the propensity of statins to interact with a variety of drugs (Williams and Feely 2002), we sought to identify as coprescribed the following:

1. Drugs known to modify the toxicity of statins by different mechanisms (pharmacokinetic or pharmacodynamic):
   Drugs increasing the toxic potential of statins by raising their plasma concentration through metabolic or transporters inhibition. The statins primarily oxidized by CYP3A4 (simvastatin, lovastatin and atorvastatin), are mostly prone to interact through these mechanisms. Interacting drugs of interest were CYP3A4 inhibitors: macrolides (erythromycin and clarithromycin), antifungal azoles, non-dihydropiridine calcium channel blockers (non-DHP CCB, verapamil and diltiazem), the antiarrhythmic amiodarone, the immunosuppressants cyclosporine and sirolimus, some HIV antivirals (indinavir, ritonavir, nelfinavir). Since fluvastatin, and to some extent rosuvastatin, are substrates of CYP2C9, inhibitors of CYP2C9 were also looked for: sulphamethoxazole, fenofibrate, some selective serotonin reuptake inhibitors (fluvoxamine, sertraline), zafirlucast.
   Drugs with similar toxicity. Fibrates and nicotinic acid are frequently given concomitantly to treat mixed hyperlipidemia at the expense of adding their own myotoxicity to that of statins.
   Drugs decreasing the effects of statins by inducing their metabolism and/or clearance. We searched for commonly used medications known to induce CYP, such as anti-seizure drugs (phenytoin, carbamazepine, oxcarbazepine) and rifampicin.
2. Drugs with narrow therapeutic window that can be affected by statins with potentially harmful consequences. The vitamin K antagonists are substrates of CYP2C9 and may compete for the enzyme with fluvastatin, but there is evidence that such interaction can also occur with other statins. Cardiac glycosides may interact with statins as competitors for membrane transporters.

We sought to verify the potential SDIs by laboratory tests, clinical symptoms or objective findings, where appropriate. Creatine kinase (CK) as a measure of myotoxicity was checked when statin potentiation was expected. A value higher than 3 times the upper limit of normal range (ULN) was considered significantly increased. The international normalized ratio (INR) reflecting the coagulation status was taken into account when interaction with coumarin anticoagulants was found. The target therapeutic range was set at 2.0÷3.0 (Ansell et al. 2008).

The severity of the potential SDIs was defined using the free online platforms for drug interactions of the web resources Medscape and Drugs.com.

The results were presented as proportions of the studied patients in the corresponding group. GraphPad Prizm software was used for the statistical evaluation of the results. Fisher’s exact test was performed to assess the difference between the proportions. Values of p < 0.05 were considered significant.

The study was approved by the Committee on Research Ethics at the Medical University of Varna. Patients’ privacy was respected and no personal information was made publicly available.

## Results

A total of 1641 patient records were examined, out of which 709 prescriptions of statins were identified, 218 at admission and 491 at discharge. These corresponded to 572 patients receiving statins. The number of prescriptions differed from the number of patients, because they fell in one of the following 3 possible categories: 1) patients admitted to hospital without a statin prescription and discharged with a statin prescription; 2) patients entering and leaving the hospital with a statin prescription, either the same, or a different one; 3) patients admitted to hospital with a statin prescription and leaving the hospital without any. Thus, for the sake of clarity, the number of patients and the number of cases (prescriptions) will be differentiated in the text that follows.

Demographic characteristics of the patients receiving statins are presented on Table 1. The vast majority were diagnosed with coronary heart disease. Men and women were equally presented, with women being older than men.

**Table 1:** **Demographic characteristics of the patients receiving statins**

Patients characteristics	Males (95% CI)	Females (95% CI)
Total number	290	282
Age	62.1 (60.80-63.32)	67.5 (66.38-68.55)*
Leading diagnosis:		
Coronary heart disease (including unstable	185	181
angina pectoris, acute myocardial infarction)		
Exacerbated chronic heart failure	56	41
Atrial fibrillation (paroxysmal or persistent)	33	34
Others (acute heart failure, ventricular arrhythmia, arterial hypertension, etc.)	16	26

*p < 0.05 vs. males.

Simvastatin, lovastatin and atorvastatin accounted for more than 80% of all statins prescribed at admission and for over 85% at discharge. Simvastatin prescriptions at discharge outnumbered almost twice those at admission. Lovastatin and atorvastatin were more frequently prescribed out of the hospital.

The total number of statin coprescriptions potentially leading to hazardous SDIs was 57 at hospital admission and 120 at discharge (Table 2). Relative to the total number of prescribed statins at admission (218) and at discharge (491), these figures gave a similar frequency (26.1 and 24.4% respectively) and corresponded to 110 patients (19.2% of all receiving a statin therapy). Simvastatin was most commonly coprescribed at discharge (59.2%), significantly more compared to admission (31.5%). Coprescriptions of atorvastatin prevailed at admission compared to discharge.

**Table 2:** **Potential statin-drug interactions (pSDIs) with all drugs of interest at hospital admission and discharge**

Statin	At hospital admission					At hospital discharge				
	Total number	% of all statins prescribed (n = 218)	% of all pSDIs (n = 57)	Number eliminated in hospital	% eliminated in hospital	Total number	% of all statins prescribed (n = 491)	% of all pSDIs (n = 120)	Number generated in hospital	% newly generated in hospital
Simvastatin	18	8.3	31.5	6	33.3	71	14.5^#^	59.2^###^	40	56
Atorvastatin	19	8.7*	33.3*	5	26.3	20	4.1	16.7	9	45
Rosuvastatin	8	3.7	14	1	12.5	16	3.3	13.3	6	37.5
Lovastatin	9	4.1	16	4	44.4	8	1.6	6.7	4	50
Fluvastatin	2	0.9	3.5	1	50	5	1.0	4.1	1	20
Pravaststin	1	0.45	1.7	-		-	-	-	-	
**Total**	**57**	**26.1**	**100**	**17**	**29.8**	**120**	**24.4**	**100**	**60**	**50** ^†^

*p < 0.05 vs. corresponding % value at hospital discharge.

^#^p < 0.05; ^###^p < 0.001 vs. corresponding % value at hospital admission.

^†^p < 0.05 vs. % pSDIs eliminated in hospital.

The number of statin coprescriptions that were eliminated during hospital stay represented approximately 30% of all coprescriptions found at admission, while at discharge, 50% of the coprescriptions were newly generated. This difference was significant as a whole (p < 0.05), and it was present as a trend with all the individual statins with the exception of fluvastatin (Table 2).

Combinations of statins with CYP3A4 inhibitors were detected at a rate of 6.4% at admission and 4.1% at hospital discharge (Table 3), the difference being insignificant. Of the CYP3A4 inhibitors, amiodarone, non-DHP CCB and macrolides were identified.

**Table 3:** **Statin coprescriptions with CYP3A4 inhibitors**

Statin	At hospital admission		At hospital discharge	
	Number	% of all statins prescribed	Number	% of all statins prescribed
Simvastatin	4	1.8	12	2.4
Atorvastatin	3	1.4	4	0.8
Rosuvastatin	1	0.45	2	0.4
Lovastatin	5	2.3 *	2	0.4
Fluvastatin	1	0.45	-	-
**Total**	**14**	**6.4**	**20**	**4.1**

*p < 0.05 vs. % lovastatin prescriptions at hospital discharge.

Amiodarone was found in 8 patients on statin therapy at entry (3.7%) and 17 patients at discharge (3.5%), of which simvastatin was involved in 12 coprescriptions, followed by atorvastatin in 6, lovastatin in 4, rosuvastatin in 2 and fluvastatin in 1 patient.

Combinations with non-DHP CCB were found in six patients at hospital entry (2.7%) involving verapamil in 5 cases (1 with simvastatin, 2 with lovastatin, 1 with atorvastatin and 1 with rosuvastatin) and diltiazem in 1 case (with simvastatin) and in two patients at discharge involving verapamil (with simvastatin and lovastatin).

Two cases of coprescription with clarithromycin were found at discharge – with simvastatin and rosuvastatin.

The potential statin interactions with the above mentioned CYP3A4 inhibitors are rated as moderately severe or severe in terms of their myotoxicity risk. We found no elevated CK in the patients receiving these combinations.

Co-treatment with CYP inducers was found in five patients – one was prescribed oxcarbazepine concomitantly with atorvastatin at admission; four received carbamazepine (3 cases) or oxcarbazepine (1 case) along with simvastatin (2 cases), atorvastatin (1 case) and fluvastatin (1 case) at discharge. The interactions of carbamazepine with simvastatin and atorvastatin are rated as moderately severe.

The combinations of statins with the vitamin K antagonist acenocoumarol identified were 25 at hospital entry and 60 at discharge (Table 4) and corresponded to 69 patients. The overall proportion was approximately the same at admission (11.5%) and at discharge (12,4%). Coprescriptions with atorvastatin predominated at admission, while those with simvastatin prevailed (not significantly) at discharge. Combinations with fluvastatin had the lowest rate. The newly generated statin-acenocoumarol coprescriptions predominated significantly (p < 0.01) over those eliminated in the hospital, the difference being entirely due to the simvastatin-acenocoumarol combinations.

**Table 4:** **Statin coprescriptions with acenocoumarol**

Statin	At hospital admission				At hospital discharge			
	Total number	% of all statins prescribed (n = 218)	Number eliminated in hospital	% eliminated in hospital	Total number	% of all statins prescribed (n = 491)	Number generated in hospital	% generated in hospital
Simvastatin	8	3.7	3	37.5	36	7.3	31	86^##^
Atorvastatin	10	4.6*	7	70	9	1.8	4	44.4
Rosuvastatin	3	1.4	0	0	9 + 1 on warfarin	2.0	7	70
Lovastatin	3	1.4	2	66.7	3	0.6	3	100
Fluvastatin	1	0.5	0	0	3	0.6	2	66.7
**Total**	**25**	**11.5**	**12**	**48**	**60**	**12.4**	**47**	**77** ^##^

*p < 0.05 vs. the corresponding % value at hospital discharge.

^##^p < 0.01 vs. % coprescriptions eliminated in hospital.

There was one patient on warfarin, coprescribed with rosuvastatin at discharge.

In 10 patients out of the 69 on statin-acenocoumarol combination, INR was found to be >3 (ranging from 3.1 to 5.1). There was no evidence of bleeding. No underlying conditions possibly affecting coagulation state (e.g. serious hepatic, renal, thyroid, etc. diseases), were found. The participating statins were simvastatin (7 cases), atorvastatin (2 cases) and rosuvastatin (1 case). Two patients, who had been previously stabilized on acenocoumarol, had their INR increased after simvastatin was added to therapy: (Pt. 1) from 2.58 to 4.0 with a simvastatin dose of 10 mg, and (Pt. 2) from 2.04 to 3.1 with a simvastatin dose of 40 mg. The first, however, also received co-amoxiclav, that could interfere with the effect of acenocoumarol. With another patient, the discontinuation of atorvastatin was followed by a decrease of INR from 3.1 to 2.4. Two more patients had concomitant prescription for co-trimoxazole and allopurinol, which are capable of potentiating acenocoumarol effect. The remaining 7 patients (10%) with high INR had no other visible reason for the observed enhanced anticoagulation except the concomitant exposure to statins. No pharmacogenetic tests were available. None of the potentially adverse interactions involved fluvastatin. The statin-acenocoumarol (warfarin) interactions are rated as either minor or moderate.

Coprescriptions with cardiac glycosides (digoxin and methyldigoxin) affected 15 patients at admission (6.9%) and 34 at discharge (6.9%), involving predominantly simvastatin, especially at discharge. No signs of digitalis or statin toxicity were found.

One patient had a rosuvastatin coprescription with ciprofibrate at admission. No other fibrate or nicotinic acid, were found prescribed concomitantly with statins.

## Discussion

There are few studies reporting the incidence of pSDIs (Einarson et al. 2002; Rätz Bravo et al. 2005; Egger et al. 2007; Stang et al. 2007; Tirkkonen et al. 2008). They all differ in their design, number and nature of interacting drugs, as well as in the patient sample size. This makes head to head comparisons difficult across existing studies, including ours.

In our study the statin most frequently coprescribed was simvastatin (Table 2). A similar tendency was found in Swiss ambulatory patients (Rätz Bravo et al. 2005). While we found no significant difference in the total proportion of pSDIs between patients at hospital admission and discharge, there was a significant increase of simvastin coprescriptions for inpatients compared to outpatients. These changes paralleled the patterns of simvastatin prescription. The percentage of newly created pSDIs for simvastatin (56%) during hospital stay was the highest among the statins as well as considerably higher than those eliminated for this statin (33%). It is worth noting that simvastatin is considered the statin most sensitive to pharmacokinetic drug interactions due to its low bioavailability (Shitara and Sugiyama 2006; Horn and Hansten 2011).

Among the statin partners in the potentially adverse combinations, the cardiovascular drugs predominated.

Amiodarone was the most commonly coprescribed CYP3A4 inhibitor that we identified, mainly with simvastatin. This combination has been associated with higher incidence of adverse effects, reported to the FDA, compared to atorvastatin and pravastatin (Alsheikh-Ali and Karas 2005). Amiodarone has been found to significantly increase the bioavailability of simvastatin, while not affecting pravastatin (Becquemont et al. 2007). Cases of rhabdomyolysis have been reported on this combination (Roten et al. 2004; Marot et al. 2011). As a measure of precaution, it has been recommended by Merck (2008), and later by FDA (2011), that simvastatin dose should not exceed 20 mg daily in patients receiving amiodarone. In our study the patients on amiodarone co-medication received no more than 20 mg of simvastatin.

The combination of statins with non-DHP CCB (moderate to potent CYP3A4 inhibitors) also exposes patients to higher risk of myotoxicity. Verapamil has been shown to increase C_max_ and AUC of simvastatin 2–3 fold (Kantola et al. 1998). Diltiazem has been found responsible for rhabdomyolysis secondary to interaction with simvastatin and atorvastatin (Kanathur et al. 2001, Lewin et al. 2002; Gladding and Pilmore 2004; Hu et al. 2011). The most recent recommendation of FDA (2011) has reconsidered the label for simvastatin dose when combined with non-DHP CCB from 20 to 10 mg. In our study relatively few patients were coprescribed verapamil and diltiazem.

Macrolides, particularly erythromycin and clarithromycin, are potent CYP 3A4 inhibitors. In combination with simvastatin they have been associated with myopathy and rhabdomyolysis and are contraindicated with this statin (Kantola et al. 1998; Lee and Maddix 2001; Molden and Andersson 2007). There was a low incidence of statin coprescriptions with macrolides in our study (two cases), obviously due to the prevalence of cardiovascular disorders.

In all the 34 cases exposed to statins plus CYP-inhibitors in the present study, no biochemical signs (CK levels >3 times ULN) reflecting damage to muscle were observed, neither were myalgia complaints of patients documented.

We identified a relatively low incidence (roughly 5%) of coprescriptions of statins and CYP inhibitors. Larger studies have found higher rates, e.g. 6% and 13% in community and hospital settings in Finland, respectively (Tirkkonen et al. 2008), and 30% among primary care patients in UK (Bakhai et al. 2012). Although the frequency of these pSDIs in our study is lower than the reported in the literature, the prevailing involvement of simvastatin is alarming, having in mind the unique vulnerability of this drug to metabolic interactions.

CYP inducers given concomitantly with statins may limit their bioavailability. Carbamazepine has been shown to substantially decrease the AUC of simvastatin (Ucar et al. 2004). In our study the patients receiving statin-carbamazepine combination had a low dose simvastatin and atorvastatin (10 mg). This may potentially compromise their therapeutic efficacy. Oxcarbazepine is a weaker CYP3A4 inducer (Andreasen et al. 2007). We have found it coprescribed with atorvastatin (unknown dose) at admission and with fluvastatin (40 mg) at discharge. The last combination is probably of no clinical relevance, since fluvastatin is not a substrate of CYP3A4.

The interaction of statins with coumarin drugs is known to be loaded with a bleeding risk. In the present study coprescription of statins with acenocoumarol was the most frequent pSDI identified. A total of 85 cases were found, affecting 69 patients.

Coumarin anticoagulants are primarily metabolized by CYP2C9, but CYP1A2 and 3A4 are also involved. The more active S-enantiomer of warfarin is metabolically dependent on CYP2C9, while CYP1A2 and 3A4 are responsible for the metabolism of the less active, R-enantiomer. Fluvastatin (Cohen et al. 2000; Sakaeda et al. 2006), and to some extent also rosuvatatin (Simonson et al. 2005; McKenney 2005; Sakaeda et al. 2006;) are expected to potentiate the effects of warfarin through competitive inhibition of CYP2C9. Indeed, most of the statin-warfarin interactions reported in the literature involve fluvastatin (Andrus 2004). However, other statins have also been associated with increase of the INR or bleeding in patients receiving warfarin, possibly interfering with its metabolism. For example, simvastatin has been suspected to be the cause of a fatal cerebral hemorrhage following a switch from atorvastatin in a patient taking warfarin, suggesting that atorvastatin and simvastatin might differ in their potential to interact with warfarin (Westergren et al. 2007). However, a recent clinical study reported that initiation of both simvastatin and atorvastatin, and not pravastatin, in chronic warfarin users, was associated with an increased risk of hospitalization for gastrointestinal bleeding (Schelleman et al. 2010). Simvastatin and lovastatin and not pravastatin have been found to decrease the plasma concentration of the main warfarin metabolite, 10-hydroxywarfarin, in patients stabilized on warfarin (Herman et al. 2006). Yu et al. (2012) reported a significant increase of INR with rosuvastatin (and not with pitavastatin) in healthy volunteers treated with warfarin at a steady-state INR of 1.5 to 2.2.

Acenocoumarol is the coumarin anticoagulant predominantly used in Bulgaria and other European countries. Information about the interactions of this drug, including with statins, is very limited compared to warfarin. For example, no information was found in the two websites where we checked drug interactions, so we used data concerning warfarin instead. It seems, however, that acenocoumarol differs in some pharmacokinetic aspects from warfarin. Thus, on the one hand, similarly to warfarin acenocoumarol is a racemic mixture of a more potent S-enantiomer (substrate of CYP2C9) and a less active R-enantiomer (oxidized by CYP3A4). But, on the other hand, due to the shorter plasma half-life of the S-enantiomer, it is the R-form that is principally responsible for the anticoagulant effect (Beinema et al. 2008). This makes the metabolic drug interactions of acenocoumarol based on competitive inhibition of CYP clinically relevant not only with fluvastatin and rosuvastatin, but also with simvastatin, atorvastatin and lovastatin (Sakaeda et al. 2006; Mondillo et al. 2005; Grau et al. 1996). Acenocoumarol differs also from phenprocoumon in its pharmacokinetic relationship to statins, since only the former has been associated with reduction of the maintenance dose in the presence of different statins (van Schie et al. 2012).

In our study, none of the statin-acenocoumarol combinations associated with elevated INR involved fluvastatin and only one involved rosuvastatin. Rather, the hazardous combinations included mainly simvastatin and atorvastatin. The few cases of coincidence between the statin addition or removal, and the corresponding changes in INR, suggest a possible causative role for statins in the altered anticoagulation. Indeed, in three patients, concomitantly given drugs – allopurinol, co-amoxiclav and co-trimoxazole, could possibly contribute to the increase of INR (Delavenne et al. 2009; Schalekamp et al. 2007). Nevertheless, the remaining proportion of 10% of the patients affected by INR elevation is still high enough to merit attention and give rise to reflection. Although the numbers are small and we cannot exclude genetic variations in the response to acenocoumarol, the results of our observation warrant attention when this drug is given concomitantly with most of the statins.

## Conclusions

1. Potential statin-drug interactions as a whole are common in patients hospitalized for cardiovascular disease. Approximately 1 out of 5 patients receiving a statin is exposed to at least one coprescription that can be associated with adverse drug reactions.
2. The proportion of pSDIs remains stable at hospital admission and hospital discharge. However, more statin coprescriptions are generated, than are eliminated, in the hospital.
3. The statin most frequently coprescribed is simvastatin – the drug also most vulnerable to drug interactions. It is recommended, therefore, that other statins, less prone to interact (pravastatin, fluvastatin and rosuvastatin), be used in settings of comedication. These drugs, however, have been found in our study to be rarely used and, correspondingly, rarely coprescribed.
4. Contrary to our expectations, pSDIs with CYP inhibitors are not very frequent. Yet, amiodarone deserves special attention as the most common hazardous partner of statins (particularly of simvastatin). Alternatives, such as pravastatin, should be preferred, when both amiodarone and a statin are indicated.
5. The acenocoumarol-statins coprescriptions are common and predominantly created in the hospital. Simvastatin is mainly involved and the combination appears to be loaded with risk of over-anticoagulation. Concomitant use of statins and acenocoumarol requires attention and close INR monitoring with adjustment of the anticoagulant dose if necessary.

## Abbreviations

CYP: Cytochrome P-450
DDIs: Drug-drug interactions
SDIs: Statin-drug interactions
pSDIs: Potential statin-drug interactions
non-DHP CCB: Non-dihydropyridine calcium channel blockers
CK: Creatin kinase
ULN: Upper limit of normal range
INR: International normalized ratio.
